# There is no six-year periodicity in tidal forcing

**DOI:** 10.1038/s41598-025-97361-0

**Published:** 2025-04-25

**Authors:** R. D. Ray, V. Viswanathan, B. F. Chao

**Affiliations:** 1 Geodesy & Geophysics Lab, NASA Goddard Space Flight Center, Greenbelt, MD, 20771 USA; 2 Planetary Geology, Geophysics, & Geochemistry Lab, NASA Goddard Space Flight Center, Greenbelt, MD, 20771 USA; 3 Center for Space Science & Tech., University of Maryland Baltimore County, Baltimore, MD, 21250 USA; 4 College of Geodesy & Geomatics, Shandong University of Science & Technology, Qingdao, China

**Keywords:** Physical oceanography, Physical oceanography

**arising from**: J. Lin and T. Qian; *Scientific Reports* https://doi.org/10.1038/s41598-019-49678-w (2019).

The El Nino–Southern Oscillation (ENSO) is one of the most important interannual modulations of Earth’s climate, and efforts to improve ENSO forecasting are most welcome. Lin and Qian^1^ (hereafter LQ19) offer an intriguing tidal connection, which, if correct, brings in a highly deterministic component that could simplify and improve forecasts. The LQ19 argument rests on the existence of a 6-year variability in the tidal forces acting on the ocean. There are several aspects to their argument that merit comment, but here we confine ourselves to the simple question of the tidal force itself. Accurate quantitative knowledge of this force has existed for over a century^2,3^, and it would be surprising to learn that an important 6-y component has only now been discovered. In fact, there is no 6-y component in the tidal force nor in the Earth’s ocean tide.

As LQ19 note, there is a 6-y periodicity in the Moon’s orbital motion which is most simply described as follows: The Moon’s orbit plane (or its ascending node) precesses with a period of 18.6 y, and its orbital ellipse (or perigee) precesses with a period of 8.85 y, both motions taken relative to the equinox. If we consider the motion of perigee relative to the moving node, its period is 6 years, simply because $(1/8.85 + 1/18.6) = 1/6.00$ (the two frequencies are added, not subtracted, because the node and perigee move in opposite directions). But does this relative motion induce a 6-y tide on Earth? It does not.

We begin by reviewing the two ways a 6-y variability in tidal forcing could possibly occur—these mechanisms do occur at other periods such as 18.6 y—and we then explain why both can be ruled out. We then examine three physical explanations that LQ19 offer in support of a 6-y tide, and we explain why each of those can also be ruled out.

## Tidal potential

 Harmonic expansions of the tide-generating potential have grown ever more comprehensive and accurate since Doodson’s work^2^ a century ago. In the much-used Cartwright-Tayler development^3^ there is no term with period 6 y; the closest are at 3.57, 8.85, and 9.31 y, all of very small amplitude. In the more recent catalog of Kudryavtsev^4^ (developed for high-precision gravimetry, not for oceanography) there are more spectral lines, and indeed one line has a period of 6.00 y. (In tidalist jargon, it is a third-degree tide with Doodson number 055.665.) Its amplitude is extremely small, well below the cutoff used by Cartwright & Tayler. We computed the corresponding equilibrium tide and found a maximum amplitude of 0.02 mm. This would be undetectable in a noisy ocean and is far too small to have any effect on climate.

## Modulations with non-linearity

 Could, however, there be modulations of harmonics (harmonic beating^5^) that give rise to a 6-y oscillation in the ocean? A familiar example is the beating of the largest lunar and solar semidiurnal tides ($\text {M}_2$ and $\text {S}_2$), leading to a 14.8-day spring-neap cycle, which, through nonlinear interactions, can lead to 14.8-d oscillations in the ocean^6^ or even the overlying atmosphere^7^. We have made a thorough search of the Kudryavtsev catalog for pairs of lines differing in frequency by 1 cycle in 6 y. There are many, but all involve very small constituents. The largest pair is within the (relatively small) $\text {M}_1$ tidal group^8^ (Doodson numbers 155.555 and 155.665, the latter a nodal sideline of a larger $\text {M}_1$ line). Examining the $\text {M}_1$ amplitudes published by Woodworth^8^ from a network of 794 tide gauges, we find the largest amplitudes anywhere of these two $\text {M}_1$ lines are 2.38 and 0.67 cm, respectively. Nonlinear interactions between these two lines could conceivably lead to 6-y oscillations in a climate variable like ocean temperature, but the effect (dependent on the product of their amplitudes) would be orders of magnitude smaller than the fortnightly interactions from the two large semidiurnal tides, whose amplitudes can reach a meter or more. As even this $\text {M}_2$-$\text {S}_2$ nonlinear effect can be difficult to detect in, say, ocean turbulence measurements^9^ or sea-surface temperatures^10^, because they are swamped by broadband, non-tidal variability, it is safe to conclude that nonlinear interactions between the much smaller $\text {M}_1$ lines are of no consequence to the climate.

This eliminates harmonic beating between two spectral lines of the tidal potential, but similar beating can also occur among a group of spectral lines^5^. Could any combination generate a significant 6-y periodicity? To limit the combinatorial searching, we restrict attention to the eight major diurnal and semidiurnal constituents—those with sufficient energy that nonlinear interactions could conceivably induce climate effects. By taking integral combinations of between two and eight constituents, we ran through 214 million combinations. The only compound period between 1 and 10 years that we found was at 4.43 y, a well-known periodicity in extreme tides^11,12^. In particular, no combination arose near 6 y.

## Lin-Qian evidence

 Nonetheless, LQ19 offer three lines of physical evidence for the existence of a 6-y tidal force. They are: (1) their analysis of lunar laser ranging (LLR) data reveals a spectral peak, purportedly in the tidal potential and arising from variations in the Earth-Moon distance with period near 6 years; (2) there is a 6-y periodicity in Earth’s angular momentum, which they assert is “anti-correlated with lunar tidal friction;” (3) an analysis of global surface temperatures done over two decades ago^13^ “demonstrates 6-year and 9-year oscillations,” supposedly driven by lunar tidal forcing. Each of these three claims warrants closer examination.

1. LQ19 state that they calculated the lunar tidal gravitational force from a time series of lunar laser ranges collected over the past half century, and from that they found a 6-y peak in the spectrum. The LLR data consist of round-trip time-of-flight measurements from an Earth station to one of the lunar surface retroreflectors. These measurements of two-way distance are modulated by a variety of orbital, rotational, geophysical, and relativistic phenomena^14^. Hence, an elaborate data reduction procedure and a dynamical model of the Moon are necessary to derive the absolute distance between the centers of the two bodies from LLR data. LQ19 give no details about the methods they used to analyze the LLR data, which raises a major concern about the neglect of standard LLR data reduction procedures in their work. Significant errors would be incurred by using uncorrected, raw LLR measurements.
  The most straightforward way to use the LLR data for examining the lunar tidal force is to calculate the Earth-Moon distance directly from an existing and accurate lunar ephemeris. State-of-art lunar ephemerides provide the necessary integrated orbits and orientation of the Moon that are constrained using LLR data and the lunar gravity field^15,16^. These fitted models are in strong agreement with the LLR data (at 1–2 cm in 1-way range). With an accurate ephemeris, the Earth-Moon distance can be computed over any desired time span and with any desired sampling, which is ideal for spectral analysis.
  Here we computed the Earth-Moon distance spectrum by employing the recent ephemeris DE440 (produced at the Jet Propulsion Laboratory^16^). One expects the spectrum to display a large peak at exactly one anomalistic month, owing to the dominating elliptical motion of the Moon. Therefore, we computed the Earth-Moon distances once per day, so that this monthly cycle is well sampled, and we computed the time series for 500 years to ensure good spectral resolution.

**Fig. 1:**
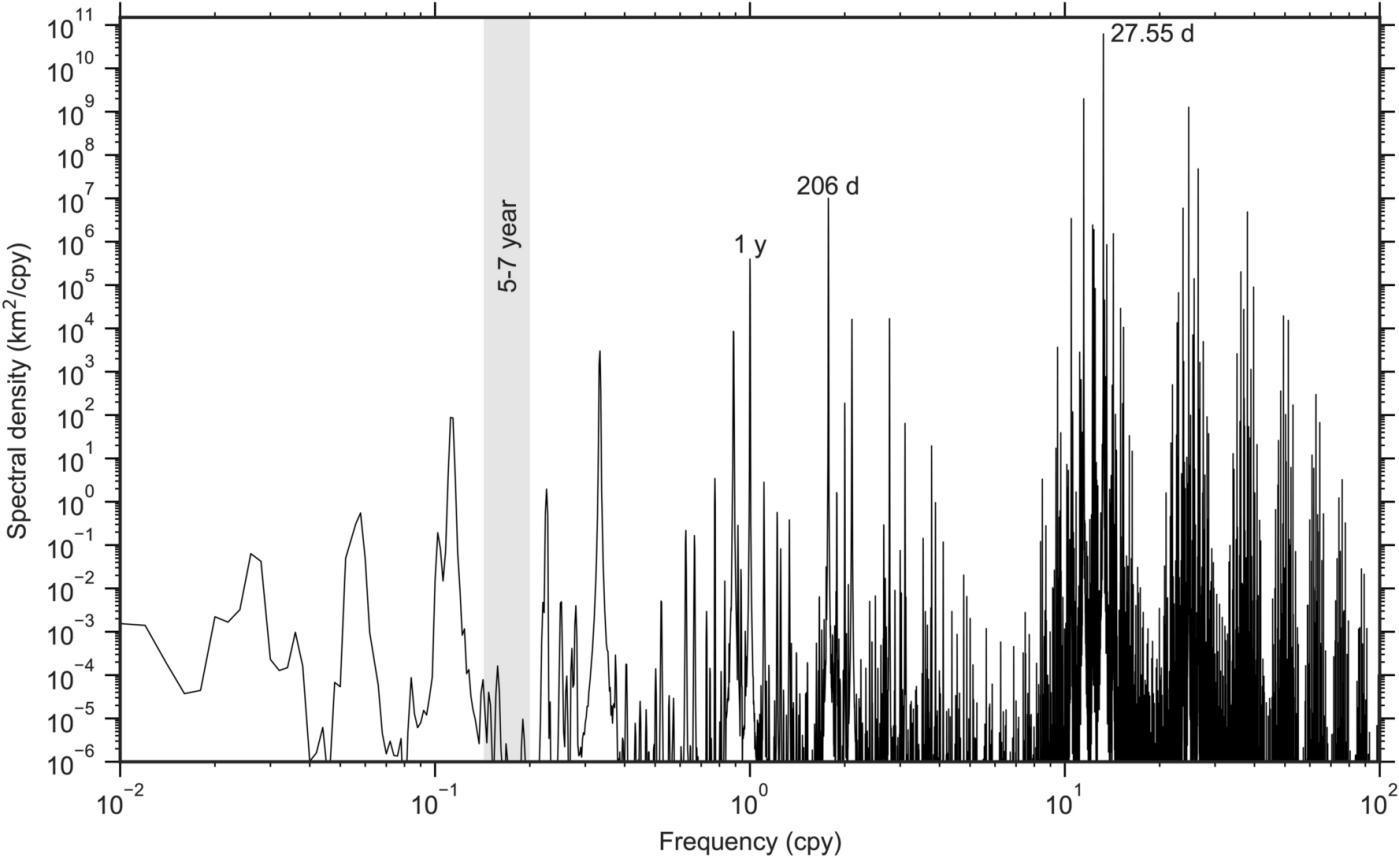
Spectrum of the earth-moon distance. The distances between Earth and Moon were computed from the DE-440 numerical ephemeris^16^. The dominant peak is at 27.5545 d (one anomalistic month). The peak at 206 d corresponds to half an evectional year. In the 5–7-y band, where LQ19 claimed to find a pronounced peak, there is a conspicuous lack of energy.
  The resulting distance spectrum is shown in Fig. 1. There are dense clusters of lines around 1 cycle/month and its higher harmonics, along with a number of longer period terms, including at periods of 206, 365, and 1095 d. Note that only the very largest of the spectral lines will lead to significant tidal effects in the ocean. For example, the primary line alone (at 27.55 d) is solely responsible for splitting the $\text {M}_2$ tidal constituent to form the $\text {N}_2$ and $\text {L}_2$ constituents^17^. In any event, in the period band 5–7 years (grey shading in the figure), where LQ19 claimed a pronounced spectral peak, there is in fact a pronounced minimum.
2. The second LQ19 argument is based on variations in the Earth’s rotation rate which they attribute to a 6-y tide. A 5.9-y oscillation in the Earth’s length of day has been previously reported in a number of studies^18,19^. This does not imply, however, a corresponding tidal force, as variations in Earth’s rotation can be induced by nontidal relative motions between its solid and fluid components. The 5.9-y oscillation is also unrelated to the tiny 6-y term in the Kudryavtsev tidal potential; being a degree-3 spherical harmonic, that term cannot directly affect Earth rotation. A growing body of work shows that the oscillation likely arises from the Earth’s core—from torsional oscillations in the Earth’s outer fluid core^20^ or gravitational coupling between the mantle and the inner core^21^ or both^22^. Geomagnetic observations place constraints on these theories, which is leading to unique insight into the Earth’s core. There is no reason to invoke a hypothetical tidal explanation for this length-of-day anomaly.
3. The third physical argument of LQ19 is based on the Keeling-Whorf hypothesis^13^ of tide-induced oscillations in surface air temperatures. Only a brief discussion is warranted here, as detailed quantitative arguments against the hypothesis are available elsewhere^23^. Keeling and Whorf had examined the times of extremes in the astronomical equilibrium tide and argued that they correlated with decadal variability in climate. The extremes they highlighted were only a few mm larger than normal perigean spring tides, and, being of semidiurnal frequency, they were “extreme” for only a few minutes at a time. Even Keeling and Whorf acknowledged (p. 8323) that it is therefore difficult to envision how such short episodic events, widely separated in time, could influence decadal climate, which is problematic enough. But it subsequently became clear that their extremes were based on only a rough proxy for the tidal force, and a more precise calculation yielded a completely different set of high-tide times^23^. In light of these problems the Keeling-Whorf hypothesis cannot be taken seriously. That LQ19 adopted that hypothesis for their own lends no weight to their argument for a 6-y tide.

## Summary

All three lines of evidence offered by LQ19 for the existence of a 6-y tidal force or a 6-y ocean tide thus fall apart. This is actually not surprising, since our knowledge of the tidal potential is quite accurate, and has been for many decades. So too is our experience with periodicities of observed tides in the ocean.

Nevertheless, there is a growing body of evidence that the ocean tides are an important source of mechanical energy for ocean mixing^24^. Moreover, there is suggestive evidence that tides can modulate climate, as the 18.6-year nodal modulation of diurnal tidal currents appears to perturb ocean temperatures in the northwest Pacific^25^. We can be sure, however, that any tide-climate mechanism does not involve a 6-year tide—either directly or through modulation of short-period tides—let alone one capable of influencing the timing of ENSO.

## Data Availability

The DE440 lunar ephemeris is available from the Jet Propulsion Laboratory; see https://ssd.jpl.nasa.gov/planets/eph_export.html. The datasets generated during and/or analysed during the current study are available from the corresponding author on reasonable request.

## References

[1] Lin, J. & Qian, T. Switch between El Nino and La Nina is caused by subsurface ocean waves likely driven by lunar tidal forcing. *Sci. Rep.* **9**, 13106 (2019). 31511602 10.1038/s41598-019-49678-w PMC6739352

[2] Doodson, A. T. The harmonic development of the tide-generating potential. *Proc. R. Soc.* **100**, 305–329 (1921).

[3] Cartwright, D. E. & Tayler, R. J. New computations of the tide-generating potential. *Geophys. J. R. Astr. Soc.* **23**, 45–74 (1971).

[4] Kudryavtsev, S. M. Improved harmonic development of the earth tide-generating potential. *J. Geodesy* **77**, 829–838 (2004).

[5] Munk, W. H., Dzieciuch, M. & Jayne, S. Millennial climate variability: Is there a tidal connection?. *J. Clim.* **15**, 370–385 (2002).

[6] Ffield, A. & Gordon, A. L. Tidal mixing signatures in the Indonesian seas. *J. Phys. Oceanogr.* **26**, 1924–1937 (1996).

[7] Ray, R. D. & Susanto, R. D. A fortnightly atmospheric ‘tide’ at Bali caused by oceanic tidal mixing in Lombok Strait. *Geosci. Lett.* **6**, 6 (2019).

[8] Woodworth, P. L. The global distribution of the M1 ocean tide. *Ocean Sci.* **15**, 431–442 (2019).

[9] Alford, M. H., Gregg, M. C. & Ilyas, M. Diapycnal mixing in the Banda Sea: Results of the first microstructure measurements in the Indonesian Throughflow. *Geophys. Res. Lett.* **26**, 2741–2744 (1999).

[10] Ray, R. D. & Susanto, R. D. Tidal mixing signatures in the Indonesian seas from high-resolution sea surface temperature data. *Geophys. Res. Lett.* **43**, 8115–8123 (2016).

[11] Pugh, D. T. & Woodworth, P. L. *Sea Level Science: Understanding Tides, Surges, Tsunamis and Mean Sea-Level Changes* (Cambridge Univ. Press, 2014).

[12] Ray, R. D. & Merrifield, M. A. The semiannual and 4.4-year modulations of extreme high tides. *J. Geophys. Res. Oceans* **124**, 5907–5922 (2019).

[13] Keeling, C. D. & Whorf, T. P. Possible forcing of global temperature by the oceanic tides. *Proc. Nat. Acad. Sci.* **94**, 8321–8328 (1997). 11607740 10.1073/pnas.94.16.8321 PMC33744

[14] Viswanathan, V. *Improving the dynamical model of the Moon using lunar laser ranging (LLR) and spacecraft data*. Phd, Université Paris (2017). Retrieved from https://tel.archives-ouvertes.fr/tel-01792665.

[15] Viswanathan, V. et al. The new lunar ephemeris INPOP17a and its application to fundamental physics. *Mon. Not. R. Astro. Soc.* **476**, 1877–1888 (2018).

[16] Park, R. S., Folkner, W. M., Williams, J. G. & Boggs, D. H. The JPL planetary and lunar ephemerides DE440 and DE441. *Astronom. J.* **161**, 105 (2021).

[17] Gerkema, T. *An Introduction to Tides* (Cambridge Univ. Press, 2019).

[18] Holme, R. & de Viron, O. Characterization and implications of intradecadal variations in length of day. *Nature* **499**, 202–205 (2013). 23846659 10.1038/nature12282

[19] Chao, B. F., Chung, W. Y., Shih, Z.-R. & Hsieh, Y. Earth’s rotation variations: a wavelet analysis. *Terra Nova* **26**, 260–264 (2014).

[20] Gillet, N., Jasult, D., Canet, E. & Fournier, A. Fast torsional waves and strong magnetic field within the earth’s core. *Nature* **465**, 74–77 (2010). 20445627 10.1038/nature09010

[21] Mound, J. E. & Buffett, B. A. Detection of a gravitational oscillation in length-of-day. *Earth Planet. Sci. Lett.* **243**, 383–389 (2006).

[22] Duan, P., Liu, G., Hu, X., Zhao, J. & Huang, C. Mechanism of the interannual oscillation in length of day and its constraint on the electromagnetic coupling at the core-mantle boundary. *Earth Planet. Sci. Lett.* **482**, 245–252 (2018).

[23] Ray, R. D. Decadal climate variability: Is there a tidal connection?. *J. Clim.* **20**, 3542–3560 (2007).

[24] Vic, C. et al. Deep-ocean mixing driven by small-scale internal tides. *Nat. Commun.* **10**, 2099 (2019). 31068588 10.1038/s41467-019-10149-5 PMC6506475

[25] Osafune, S. & Yasuda, I. Remote impacts of the 18.6 year period modulation of localized tidal mixing in the North Pacific. *J. Geophys. Res.* **118**, 3128–3137 (2013).

